# Utility of serum nuclear factor erythroid 2-related factor 2 as a potential prognostic biomarker of severe traumatic brain injury in adults: A prospective cohort study

**DOI:** 10.3389/fneur.2022.1013062

**Published:** 2022-11-01

**Authors:** Xin-Jiang Yan, Cheng-Peng Zhan, Yao Lv, Dan-Dan Mao, Ri-Cheng Zhou, Yong-Min Xv, Guo-Feng Yu

**Affiliations:** Department of Neurosurgery, The Quzhou Affiliated Hospital of Wenzhou Medical University, Quzhou People's Hospital, Quzhou, China

**Keywords:** biomarkers, nuclear factor erythroid 2-related factor 2, severity, prognosis, traumatic brain injury

## Abstract

**Objective:**

Nuclear factor erythroid 2-related factor 2 (Nrf2) may harbor endogenous neuroprotective role. We strived to ascertain the prognostic significance of serum Nrf2 in severe traumatic brain injury (sTBI).

**Methods:**

This prospective cohort study included 105 controls and 105 sTBI patients, whose serum Nrf2 levels were quantified. Its relations to traumatic severity and 180-day overall survival, mortality, and poor prognosis (extended Glasgow Outcome Scale score 1–4) were discerned using multivariate analysis.

**Results:**

There was a substantial enhancement of serum Nrf1 levels of patients (median, 10.9 vs. 3.3 ng/ml; *P* < 0.001), as compared to controls. Serum Nrf2 levels were independently correlative to Rotterdam computed tomography (CT) scores (ρ = 0.549, *P* < 0.001; t = 2.671, *P* = 0.009) and Glasgow Coma Scale (GCS) scores (ρ = −0.625, *P* < 0.001; t = −3.821, *P* < 0.001). Serum Nrf2 levels were significantly higher in non-survivors than in survivors (median, 12.9 vs. 10.3 ng/ml; *P* < 0.001) and in poor prognosis patients than in good prognosis patients (median, 12.5 vs. 9.4 ng/ml; *P* < 0.001). Patients with serum Nrf2 levels > median value (10.9 ng/ml) had markedly shorter 180-day overall survival time than the other remainders (mean, 129.3 vs. 161.3 days; *P* = 0.002). Serum Nrf2 levels were independently predictive of 180-day mortality (odds ratio, 1.361; *P* = 0.024), overall survival (hazard ratio, 1.214; *P* = 0.013), and poor prognosis (odds ratio, 1.329; *P* = 0.023). Serum Nrf2 levels distinguished the risks of 180-day mortality and poor prognosis with areas under receiver operating characteristic curve (AUCs) at 0.768 and 0.793, respectively. Serum Nrf2 levels > 10.3 ng/ml and 10.8 ng/ml discriminated patients at risk of 180-day mortality and poor prognosis with the maximum Youden indices of 0.404 and 0.455, respectively. Serum Nrf2 levels combined with GCS scores and Rotterdam CT scores for death prediction (AUC, 0.897; 95% CI, 0.837–0.957) had significantly higher AUC than GCS scores (*P* = 0.028), Rotterdam CT scores (*P* = 0.007), or serum Nrf2 levels (*P* = 0.006) alone, and the combination for poor outcome prediction (AUC, 0.889; 95% CI, 0.831–0.948) displayed significantly higher AUC than GCS scores (*P* = 0.035), Rotterdam CT scores (*P* = 0.006), or serum Nrf2 levels (*P* = 0.008) alone.

**Conclusion:**

Increased serum Nrf2 levels are tightly associated with traumatic severity and prognosis, supporting the considerable prognostic role of serum Nrf2 in sTBI.

## Introduction

Severe traumatic brain injury (sTBI), one of the most severe traumas, is characterized by high morbidity and mortality (1). Its pathological mechanisms involve primary brain injury and secondary brain injury (2). Its molecular processes are very complex and include inflammation, oxidative stress, and apoptosis (3). Clinically, the Glasgow Coma Scale (GCS) and Rotterdam computed tomography (CT) scale are the two frequently used severity indicators, which are strongly associated with prognosis of TBI (4, 5). Recently, researchers have paid extensive attention to biomarkers with respect to severity assessment and prognosis prediction of TBI (6–8).

Nuclear factor erythroid 2-related factor 2 (Nrf2) is a kind of recently discovered nuclear factor for antioxidant response element (ARE)-regulated genes, which is also a key regulator of endogenous inducible defense systems in the body (9). Acute oxidative stress or inflammation can greatly upregulate expressions of Nrf2 in neurons of animals or humans under some pathological conditions, such as TBI, intracerebral hemorrhage, and ischemic stroke (10–14). Accumulating experimental data have shown that Nrf2 may confer brain-protective effects *via* regulating the expression of genes coding antioxidant, anti-inflammatory, and detoxifying proteins (10–13). Thus, it is postulated that circulating Nrf2 may serve as a promising biochemical maker of acute brain injury. Our study aimed to explore the prognostic value of serum Nrf2 in a group of sTBI patients.

## Materials and methods

### Study design and participant selection

Patients diagnosed with sTBI (GCS scores < 9) were enrolled into this prospective observational study, which was performed at our hospital from February 2017 to May 2021. The inclusion criteria were as follows: (1) age ≥ 18 years, (2) injury severity score < 9 in non-cranial aspects, (3) blunt trauma, and (4) hospital admission within 12 h after trauma. The exclusion criteria were as follows: (1) previous neurological diseases, such as stroke, intracranial tumors, cerebral aneurysms, vascular malformations, and severe head trauma, and (2) other specific conditions or diseases, e.g., severe infections within recent a month, autoimmune diseases, pregnancies, severe hepatic, renal or cardiac diseases, and malignancies. In addition, controls were a group of healthy non-patient volunteers recruited from May 2020 to May 2021 at our hospital, who were free of some chronic diseases, such as hypertension, diabetes mellitus, and coronary heart disease, and were normal in routine tests, e.g., blood leucocyte count, blood hemoglobin levels, and blood platelet count. This study followed the ethical guidelines of the Declaration of Helsinki, and the approval for the protocol of this study was acquired from the institutional review committee at our hospital. All the relatives of patients and controls themselves gave informed written consent prior to inclusion in the study.

### Demographic, clinical, and radiological information

We gathered demographic information, past medical history, cigarette smoking history, and alcohol drinking history. Baseline GCS scores were recorded to reflect clinical severity of head trauma. The radiological parameters were determined by research personnel blinded to clinical data using the first available head CT scan. Positive radiological appearances included abnormal cisterns, midline shift, epidural hematoma, subdural hematoma, subarachnoid hemorrhage, intraventricular hemorrhage, intracerebral hematoma, brain contusion, and pneumocephalus. Rotterdam CT scores were calculated to assess radiological severity of head trauma. A functional outcome was evaluated using eight-grade extended Glasgow Outcome Scale (GOSE) at 180 days following trauma. GOSE was dichotomized into poor outcome (1–4 points) and good outcome (4–6 points) (15).

### Measurement of serum Nrf2 levels

Blood samples were collected from all study participants, including patients and controls. The blood was centrifuged, and then, serum samples were immediately preserved at −80°C until required for further analysis. The same technician, who was blinded to clinical data, performed measurement of serum Nrf2 levels using a commercially available DNA-binding enzyme-linked immunosorbent assay (ELISA) (TransAM Nrf2, Active Motif, Carlsbad, CA, USA) as per the manufacturer's protocol. In brief, the samples were incubated for 1 h in a pre-coated 96-well-plate, followed by primary antibody incubation for 1 h. After incubation, all the wells were washed and incubated with horseradish peroxidase-conjugated antibody for 1 h, followed by trimethylbenzene substrate for 15 min in the dark. The reaction was stopped by the addition of stop solution, and the plate was read at 450 nm on a microplate reader (Multiskan GO; Thermo Fisher Scientific, Waltham, MA, USA). Each sample was in duplicate determined, and the results were averaged for further analysis.

### Statistical analysis

Receiver operating characteristic (ROC) curve analysis was carried out using MedCalc statistical software version 17.4 (MedCalc Software, Mariakerke, Belgium), and other data analyses were performed utilizing the SPSS 20.0 statistical package (SPSS Inc., Chicago, Illinois, USA). Qualitative variables were shown as frequencies (proportions). Categorical data were compared between two groups using Pearson's Chi-square test or Fisher's exact test as appropriate. Quantitative data, which were tested for normal distribution, were presented as means (standard deviations, SDs) if normally distributed or medians (percentages 25th−75th) if non-normally distributed. Statistical methods of intergroup comparisons of quantitative data included the independent *t*-test for normally distributed data and the Mann–Whitney test for non-normally distributed data. Using the Kruskal–Wallis H test, serum Nrf2 levels were compared among multiple groups divided by GCS scores and Rotterdam CT scores. Serum Nrf2 levels were dichotomized based on its median value, and 180-day overall survival time was compared using the log-rank test. Under ROC curve, the prognostic predictive capability was assessed, and area under ROC curve (AUC) and the corresponding 95% confidence interval (CI) were yielded. 180-day death and poor prognosis were regarded as two dependent variables. The two multivariate binary logistic regression models were established, and significant variables in univariate analysis were forced to identify independent predictors of 180-death and poor prognosis. Associations were reported as odds ratio (ORs) and 95% CIs. Univariate Cox's proportional hazard analysis was performed to screen variables, which were substantially related to 180-day overall survival, and afterward, multivariate Cox's proportional hazard analysis was performed to discern independent predictors. Hazard ratio (HRs) and 95% CIs were reported for showing associations. Bivariate correlations were analyzed using the Spearman test, and thereafter, multivariate linear regression model was configured to ascertain variables, which were independently correlated with serum Nrf2 levels. For all tests, the two-sided *P* < 0.05 were considered statistically significant.

## Results

### Patient selection and participant characteristics

**Figure 1:**
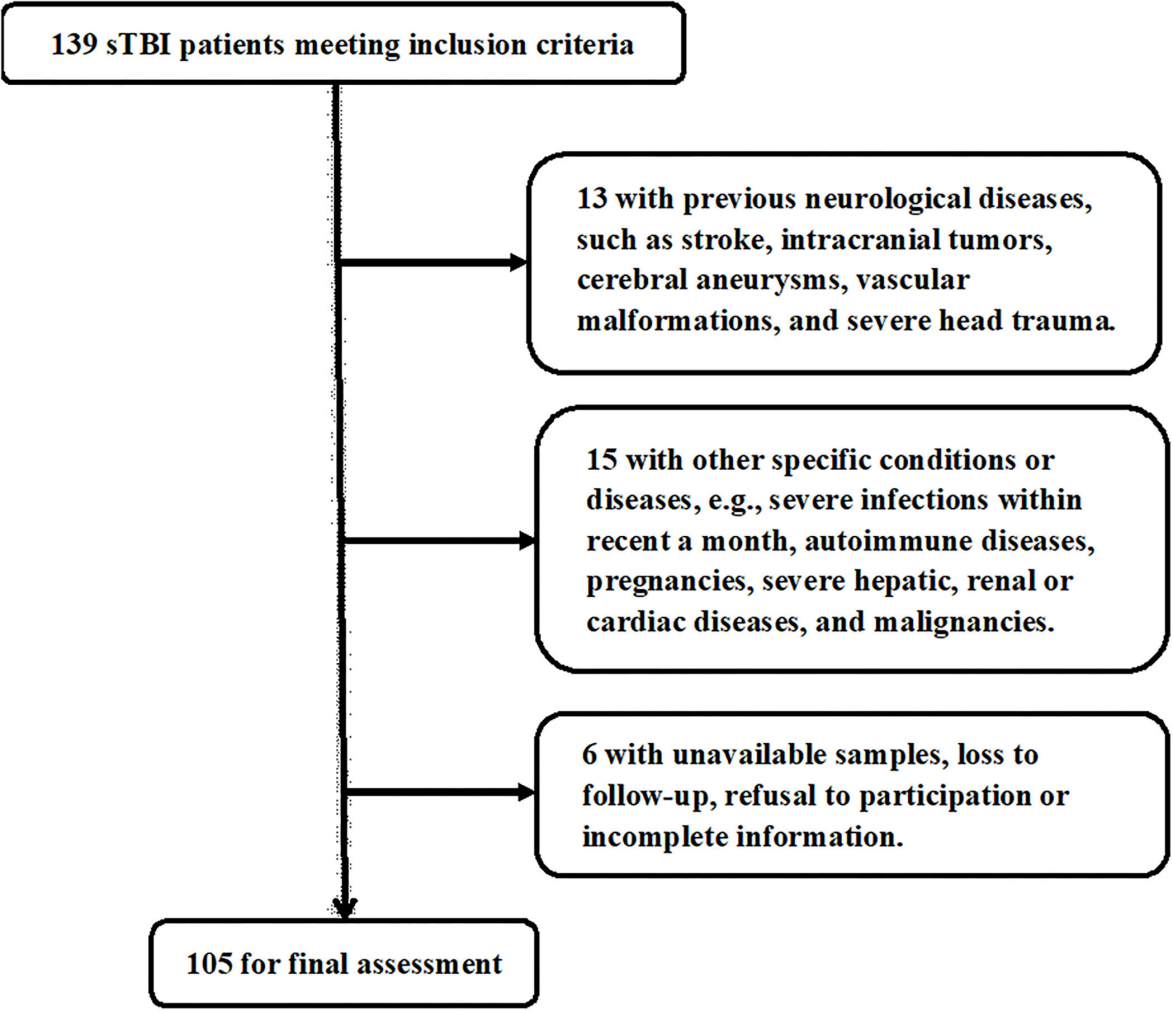
Flowchart for selecting eligible patients with severe traumatic brain injury. Among 139 patients with severe traumatic brain injury, who conformed to preset inclusion criteria, a total of 34 patients were excluded, and 105 patients were eventually assessed for further analysis. sTBI denotes severe traumatic brain injury.

In Figure 1, a total of 139 sTBI patients fitted the inclusion criteria, 34 patients were thereafter removed from this study based on the exclusion criteria, and eventually 105 patients were analyzed for epidemiological investigation. In addition, a group of controls, including 105 healthy individuals, were enrolled. Patients were composed of 60 males and 45 females and were aged from 18 to 78 years (mean, 42.3 years; SD, 14.2 years). And controls were comprised of 54 males and 51 females and were aged from 18 to 77 years (mean, 43.8 years; SD, 13.8 years). Age and gender percentage were not significantly different between controls and patients (both *P* > 0.05).

Among this cohort of patients, 32 were cigarette smokers, 33 consumed alcohol, 17 suffered from hypertension, 12 presented with diabetes mellitus, and 17 were inflicted with hyperlipidemia. Hospital admission time ranged from 0.5 to 12.0 h after trauma (median, 4.8 h; percentiles 25–75th, 3.5–6.3 h). Blood-sampling time ranged from 1.0 to 14.5 h (median, 6.3 h; percentiles 25–75th, 4.5–8.1 h) following trauma. Traumas of 52 cases were caused by automobile/motorcycle; those of 44 cases, by fall/jump; and those of 9 cases, by others. GCS score ranged from 3 to 8 (median, 5; upper-lower quartiles, 4–6). Rotterdam CT score ranged from 3 to 6 (median, 4; upper-lower quartiles, 4–5). The mean systolic and diastolic arterial pressures were 125.7 mmHg (SD, 29.6 mmHg; range, 75–182 mmHg) and 74.7 mmHg (SD, 17.1 mmHg; range, 45–109 mmHg), respectively. Blood glucose levels ranged from 5.2 to 20.7 mmol/l (median, 8.8 mmol/l; lower–upper quartiles, 7.2–11.4 mmol/l). Blood leucocyte count ranged from 3.2 to 16.1 × 10^9^/l (median, 7.5 × 10^9^/l; lower–upper quartiles, 6.0–10.0 × 10^9^/l).

### Change of serum Nrf2 levels and its relation to trauma severity

**Figure 2:**
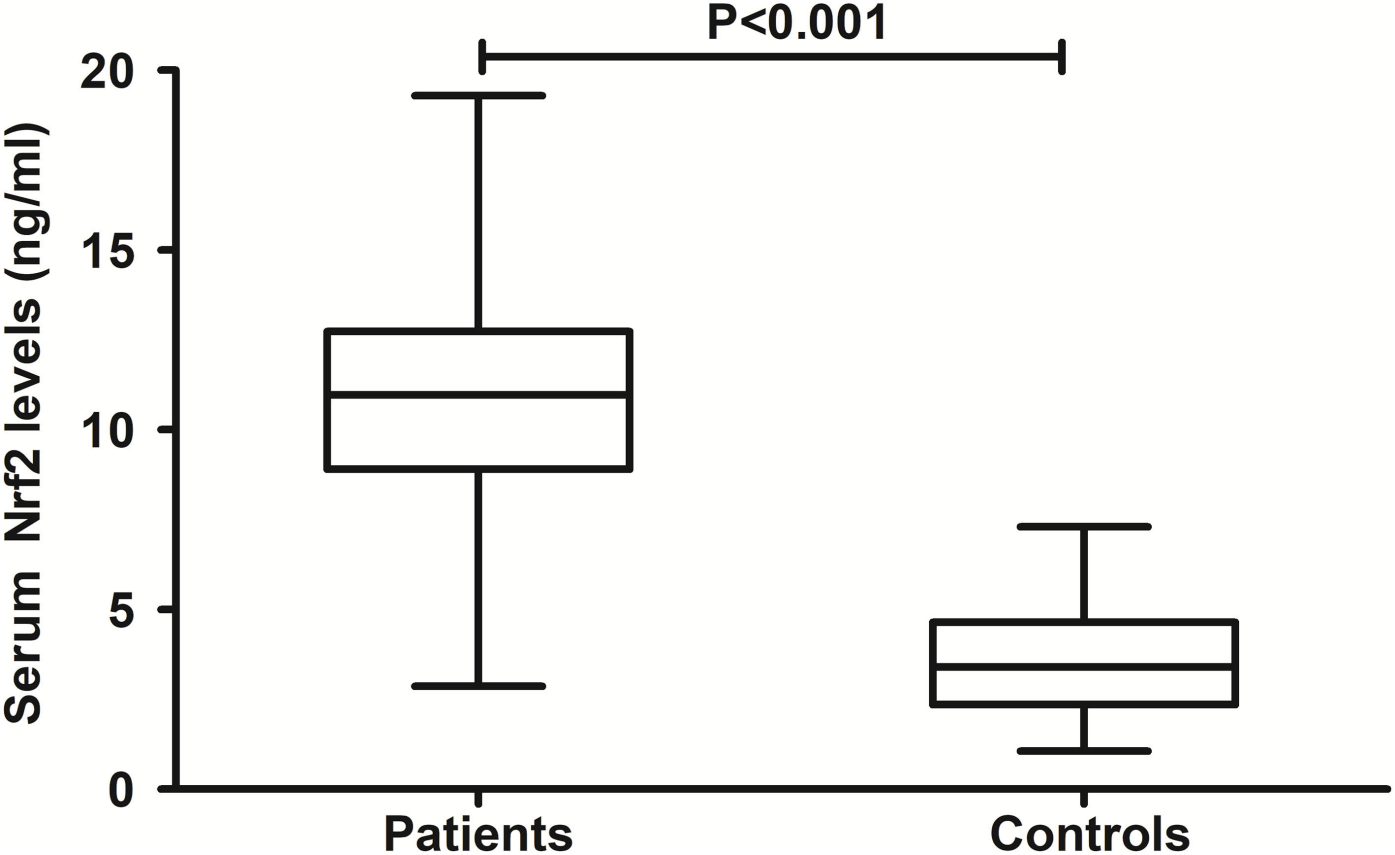
Comparison of serum nuclear factor erythroid 2-related factor 2 levels between controls and patients with severe traumatic brain injury. Patients with severe traumatic brain injury had significantly higher serum nuclear factor erythroid 2-related factor 2 levels than healthy controls (*P* < 0.001). Nrf2 means nuclear factor erythroid 2-related factor 2.

**Figure 3:**
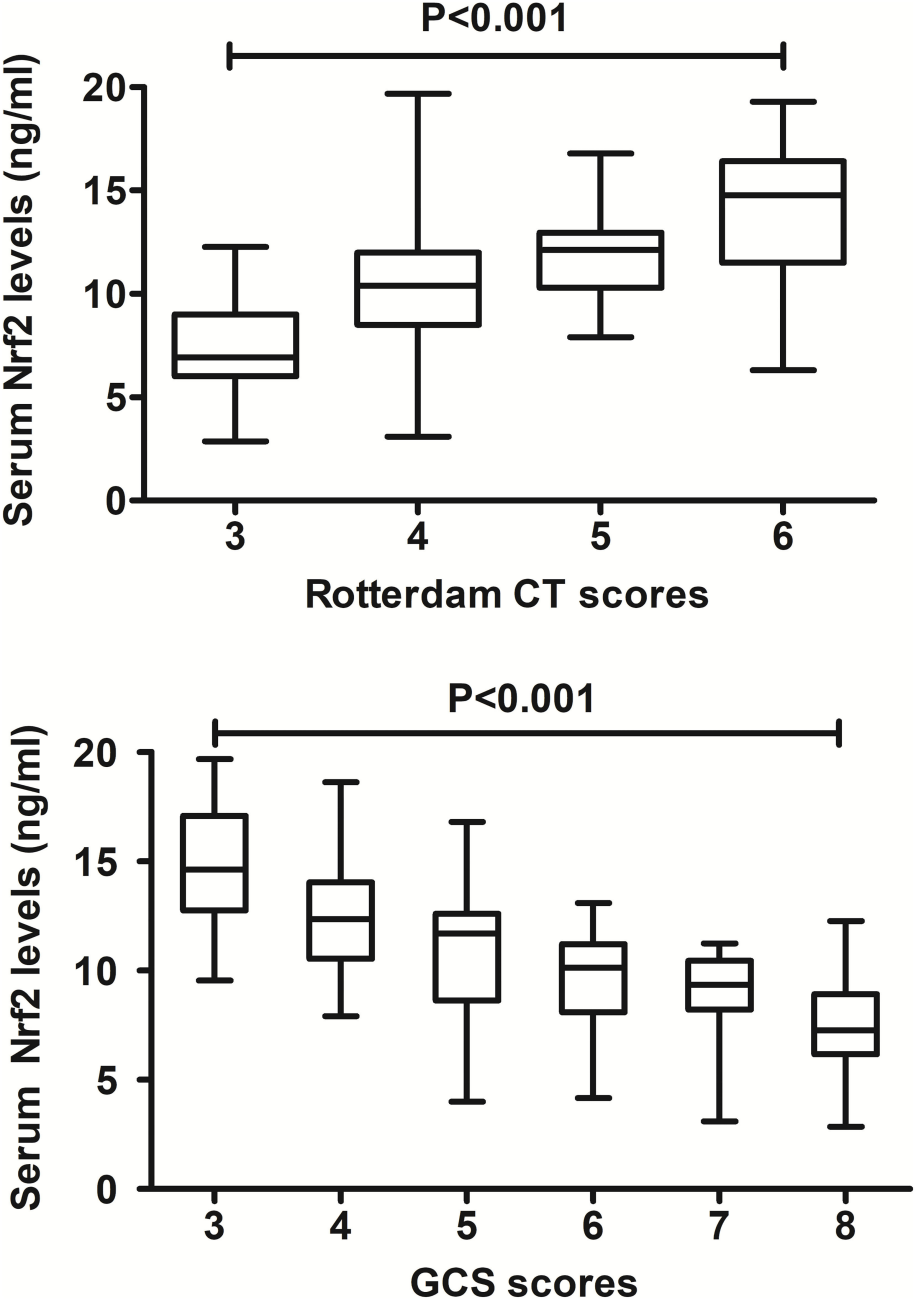
Relationship between serum nuclear factor erythroid 2-related factor 2 levels and traumatic severity in patients with severe traumatic brain injury. There were substantial differences in terms of serum nuclear factor erythroid 2-related factor 2 levels among subgroups divided by Rotterdam computed tomography scores and Glasgow Coma Scale scores in patients with severe traumatic brain injury (both *P* < 0.001). Nrf2 indicates nuclear factor erythroid 2-related factor 2; CT, computed tomography; GCS, Glasgow Coma Scale.

In Figure 2, there was a substantial elevation of serum Nrf2 levels in patients, as compared to controls (*P* < 0.001). Just as listed in Table 1, variables, which were intimately correlated with serum Nrf2 levels of patients using Spearman's test, were age, diabetes mellitus, GCS scores, and Rotterdam CT scores. Using multivariate logistic linear analysis, variables, which retained independently correlated with serum Nrf2 levels, were GCS scores (t = −3.821, *P* < 0.001) and Rotterdam CT scores (t = 2.671, *P* = 0.009). Alternatively, 14, 25, 29, 15, 10, and 12 patients had GCS scores 3, 4, 5, 6, 7, and 8, respectively. Moreover, 11, 50, 25, and 19 patients had Rotterdam CT scores 3, 4, 5, and 6, respectively. In Figure 3, serum Nrf2 levels were significantly lowest in patients with Rotterdam CT score 3, followed by Rotterdam CT scores 4 and 5, and were substantially highest in those with Rotterdam CT score 6 (*P* < 0.001). Similarly, there were markedly highest serum Nrf2 levels in patients with GCS score 3, followed by GCS scores 4, 5, 6, and 7, and pronouncedly lowest in those with GCS score 8 (*P* < 0.001, Figure 3).

**Table 1:** Factors correlated with serum nuclear factor erythroid 2-related factor 2 levels after severe traumatic brain injury.

	**ρ**	***P-*value**
Gender (male/female)	0.093	0.344
Age (y)	0.214	0.028
Current cigarette smoking	0.139	0.158
Alcohol abuse	0.091	0.354
Hypertension	0.046	0.638
Diabetes mellitus	0.211	0.030
Hyperlipidemia	0.050	0.610
Hospital admission time (h)	−0.098	0.319
Blood-sampling time (h)	−0.100	0.308
Traumatic causes	−0.157	0.110
GCS scores	−0.625	< 0.001
Rotterdam CT scores	0.549	< 0.001
Systolic arterial pressure (mmHg)	−0.164	0.095
Diastolic arterial pressure (mmHg)	−0.075	0.448
Blood glucose levels (mmol/l)	0.171	0.082
Blood leucocyte count ( × 10^9^/l)	0.137	0.165

Correlations were analyzed using Spearman's test. CT, computerized tomography; GCS, Glasgow Coma Scale.

### Relationship between serum Nrf2 levels and death within post-traumatic 180 days

**Figure 4:**
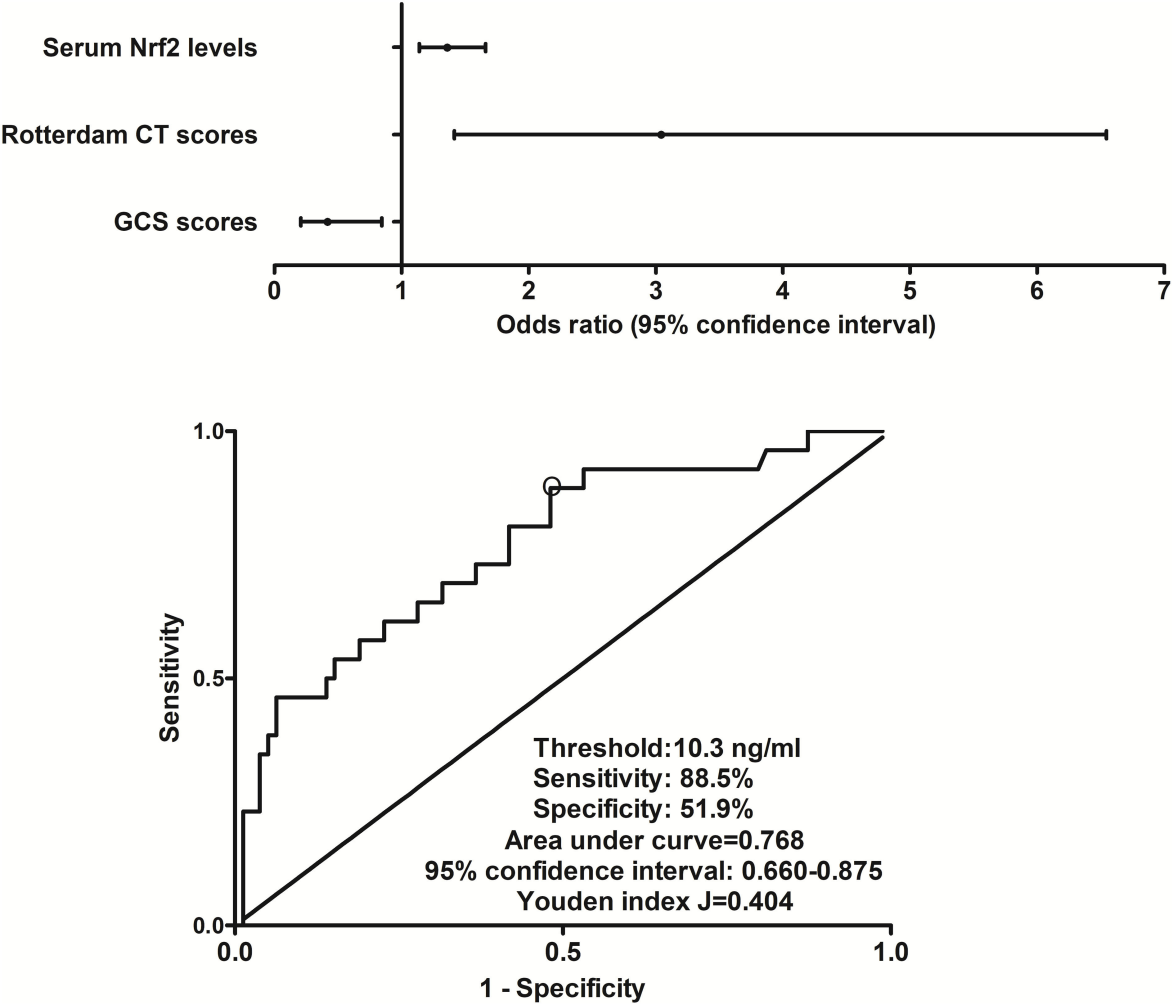
Relationship between serum nuclear factor erythroid 2-related factor 2 levels and 180-day death after severe traumatic brain injury. Serum nuclear factor erythroid 2-related factor 2 levels, Rotterdam computed tomography scores, and Glasgow Coma Scale scores were the three independent predictors of 180-day death after severe traumatic brain injury (all *P* < 0.05). Serum nuclear factor erythroid 2-related factor 2 levels were significantly predictive of 180-day death under receiver operating characteristic curve (*P* < 0.001), and using Youden's method, an optimal value of nuclear factor erythroid 2-related factor 2 levels was chosen, which produced medium–high sensitivity and specificity values for death prediction. Nrf2 indicates nuclear factor erythroid 2-related factor 2; CT, computed tomography; GCS, Glasgow Coma Scale.

In this group of sTBI patients, 180-day mortality was 24.8% (26/105). Non-survivors were significantly older than survivors, GCS scores were substantially lower, and Rotterdam CT scores were markedly higher in the dead than in the alive, and serum Nrf2 levels and blood glucose levels were profoundly raised in non-surviving patients, as compared to surviving ones (all *P* < 0.05, Table 2). The preceding variables were forced into the binary logistic regression model, and as a subsequence, GCS scores, Rotterdam CT scores, and serum Nrf2 levels emerged as the three independent factors of 180-day death (all *P* < 0.05, Figure 4). Also, serum Nrf2 levels significantly distinguished the risk of 180-day death, and using Youden's method, an optimal value of serum Nrf2 levels was selected, which generated medium–high sensitivity and specificity values for death prediction (Figure 4). In addition, its death predictive ability was equivalent to those of GCS scores (AUC, 0.836; 95% CI, 0.762-0.909; *P* = 0.177) and Rotterdam CT scores (AUC, 0.824; 95% CI, 0.735–0.914; *P* = 0.325). Serum Nrf2 levels combined with GCS scores and Rotterdam CT scores (AUC, 0.897; 95% CI, 0.837–0.957) had significantly higher AUC than GCS scores (*P* = 0.028), Rotterdam CT scores (*P* = 0.007), or serum Nrf2 levels (*P* = 0.006) alone.

**Table 2:** Factors associated with 180-day mortality after severe traumatic brain injury.

	**The dead**	**The alive**	***P-*value**
Gender (male/female)	16/10	44/35	0.602
Age (y)	47.6 ± 16.2	40.5 ± 13.1	0.026
Current cigarette smoking	11 (42.3%)	21 (26.6%)	0.131
Alcohol abuse	11 (42.3%)	22 (27.9%)	0.168
Hypertension	7 (26.9%)	10 (12.7%)	0.123
Diabetes mellitus	5 (19.2%)	7 (8.9%)	0.166
Hyperlipidemia	5 (19.2%)	12 (15.2%)	0.759
Hospital admission time (h)	4.5 (2.9–5.5)	5.1 (3.9–6.5)	0.117
Blood-sampling time (h)	5.2 (3.6–7.5)	6.8 (4.9–8.4)	0.100
Traumatic causes			0.391
Automobile/motorcycle	15	37	
Fall/jump	8	36	
Others	3	6	
GCS scores	4 (3–4)	5 (5–7)	< 0.001
Rotterdam CT scores	6 (5–6)	4 (4–5)	< 0.001
Systolic arterial pressure (mmHg)	126.2 ± 29.2	125.6 ± 29.8	0.925
Diastolic arterial pressure (mmHg)	76.0 ± 17.4	73.6 ± 17.1	0.532
Blood glucose levels (mmol/l)	10.4 (8.6–12.6)	8.4 (6.8–10.9)	0.003
Blood leucocyte count ( × 10^9^/l)	8.5 (6.2–10.8)	7.3 (5.8–9.6)	0.478
Serum Nrf2 levels (ng/ml)	12.9 (11.0–15.2)	10.3 (8.3–12.4)	< 0.001

Data were shown as mean ± standard deviation, median (25–75th percentiles), or count (percentage) where appropriate. Statistical methods for intergroup comparison included the t-test, Mann–Whitney U test, Pearson Chi-square test, or Fisher's exact test as appropriate. CT, computerized tomography; GCS, Glasgow Coma Scale; Nrf2, nuclear factor erythroid 2-related factor 2.

### Relationship between serum Nrf2 levels and 180-day overall survival after trauma

**Figure 5:**
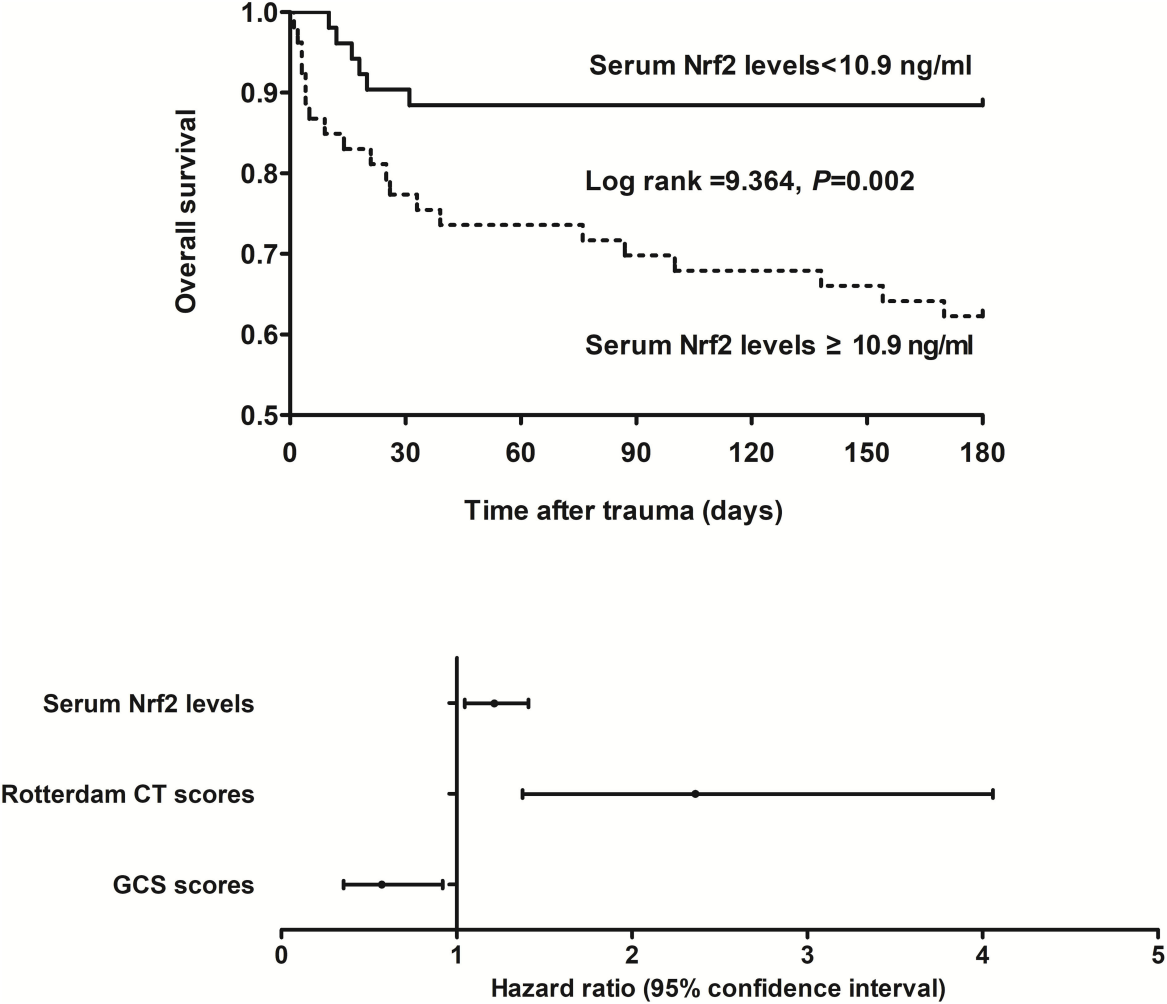
Relationship between serum nuclear factor erythroid 2-related factor 2 levels and 180-day overall survival after severe traumatic brain injury. Serum nuclear factor erythroid 2-related factor 2 levels, Rotterdam computed tomography scores, and Glasgow Coma Scale scores were independently associated with 180-day overall survival after severe traumatic brain injury (all *P* < 0.05). Patients with serum nuclear factor erythroid 2-related factor 2 levels ≥ 10.9 ng/ml (median value) displayed substantially shorter 180-day overall survival time than the other remainders (*P* < 0.01). Nrf2 indicates nuclear factor erythroid 2-related factor 2; CT, computed tomography; GCS, Glasgow Coma Scale.

This cohort of sTBI patients had the mean overall survival time of 145.2 days (95% CI, 132.6–157.7 days) during 180-day follow-up after head trauma. In accordance with the median value of serum Nrf2 levels (namely, 10.9 ng/ml), all patients were dichotomized. In Figure 5, patients with serum Nrf2 levels ≥ 10.9 ng/ml exhibited profoundly shorter 180-day overall survival time, as compared to those with serum Nrf2 levels < 10.9 ng/ml (*P* < 0.01). Just as displayed in Table 3, the factors, which were significantly related to 180-day overall survival following trauma, were age, GCS scores, Rotterdam CT scores, blood glucose levels, and serum Nrf2 levels using univariate Cox's proportional hazard model (all *P* < 0.05). Furthermore, the afore-mentioned significant pertinent variables were incorporated in multivariate Cox's proportional hazard model, and sequentially, it was confirmed that GCS score, Rotterdam CT score, and serum Nrf2 levels were independently associated with 180-day overall survival after head trauma (all *P* < 0.05, Figure 5).

**Table 3:** Factors associated with 180-day overall survival after severe traumatic brain injury.

	**Hazard ratio**	**95% CI**	***P-*value**
Gender (male/female)	1.218	0.553–2.685	0.624
Age (y)	1.031	1.003–1.059	0.028
Current cigarette smoking	1.688	0.775–3.675	0.187
Alcohol abuse	1.678	0.770–3.653	0.193
Hypertension	2.089	0.878–4.972	0.096
Diabetes mellitus	2.116	0.798–5.612	0.132
Hyperlipidemia	1.267	0.478–3.359	0.635
Hospital admission time (h)	0.904	0.772–1.057	0.207
Blood-sampling time (h)	0.899	0.783–1.034	0.136
Traumatic causes			
Automobile/motorcycle	Reference	
Fall/jump	0.798	0.231–2.757	0.724
Others	0.506	0.134–1.907	0.314
GCS scores	0.374	0.249–0.561	< 0.001
Rotterdam CT scores	3.783	2.315–6.182	< 0.001
Systolic arterial pressure (mmHg)	1.001	0.988–1.014	0.921
Diastolic arterial pressure (mmHg)	1.008	0.985–1.032	0.484
Blood glucose levels (mmol/l)	1.120	1.025–1.225	0.013
Blood leucocyte count ( × 10^9^/l)	1.059	0.929–1.206	0.394
Serum Nrf2 levels (ng/ml)	1.324	1.179–1.488	< 0.001

Associations were reported as hazard ratios using univariate Cox's proportional hazard regression analysis. CT, computerized tomography; GCS, Glasgow Coma Scale; Nrf2, nuclear factor erythroid 2-related factor 2; 95% CI, 95% confidence interval.

### Relationship between serum Nrf2 levels and 180-day poor outcome after trauma

**Figure 6:**
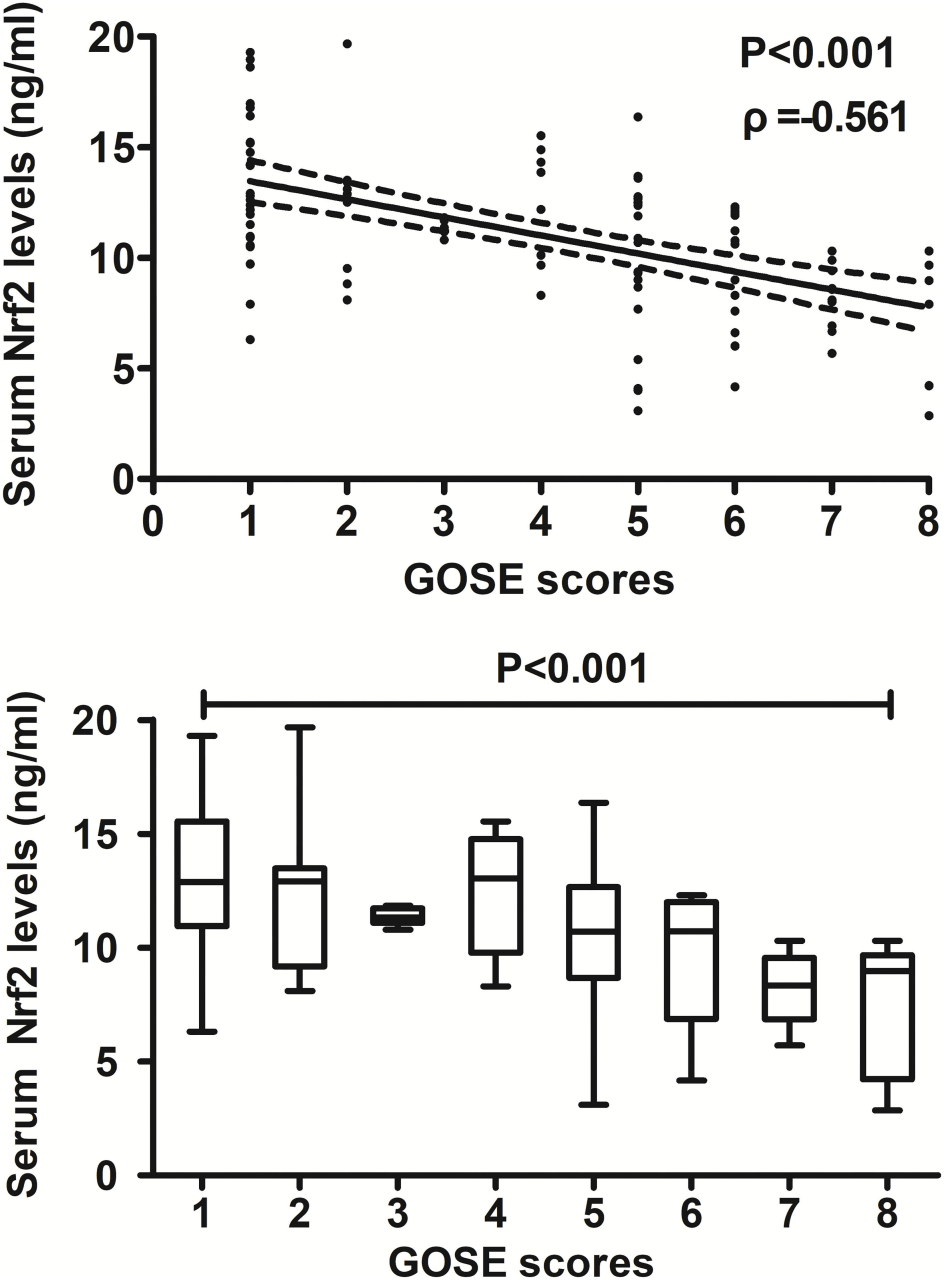
Relationship between serum nuclear factor erythroid 2-related factor 2 levels and 180-day extended Glasgow Outcome Scale scores after severe traumatic brain injury. Serum nuclear factor erythroid 2-related factor 2 levels were significantly and inversely correlated with extended Glasgow Outcome Scale scores (*P* < 0.001) and were substantially different among subgroups divided by extended Glasgow Outcome Scale scores (*P* < 0.001). Nrf2 indicates nuclear factor erythroid 2-related factor 2; GOSE, extended Glasgow Outcome Scale.

**Figure 7:**
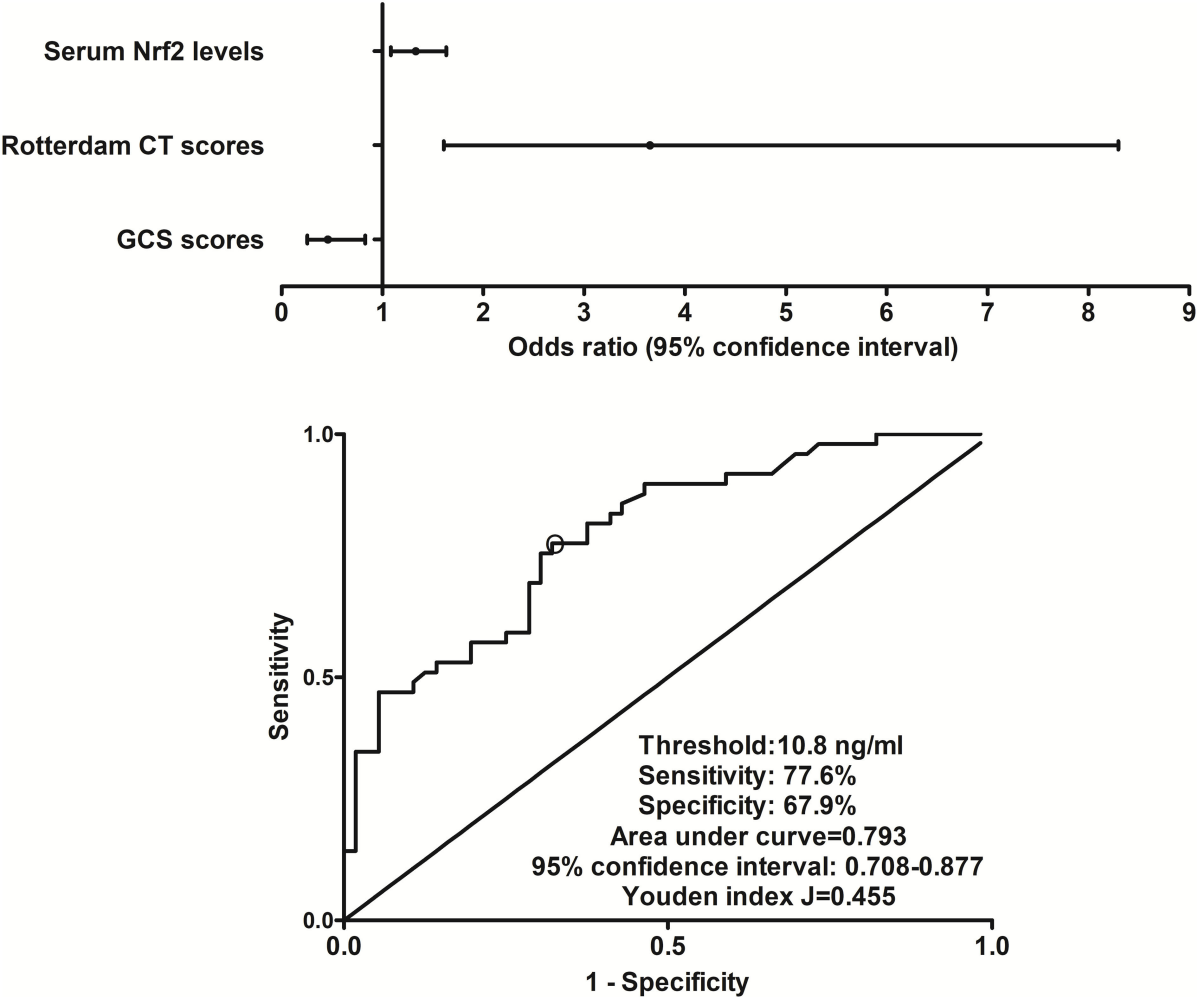
Relationship between serum nuclear factor erythroid 2-related factor 2 levels and 180-day poor outcome after severe traumatic brain injury. Serum nuclear factor erythroid 2-related factor 2 levels, Rotterdam computed tomography scores, and Glasgow Coma Scale scores remained as the three independent predictors of 180-day poor outcome (extended Glasgow Outcome Scale scores of 1–4) following severe traumatic brain injury (all *P* < 0.05). Serum nuclear factor erythroid 2-related factor 2 levels markedly discriminated patients at risk of 180-day poor outcome based on receiver operating characteristic curve (*P* < 0.001), and using Youden's method, an optimal criterion of nuclear factor erythroid 2-related factor 2 levels was selected, which yielded medium–high sensitivity and specificity values for poor outcome prediction. Nrf2 indicates nuclear factor erythroid 2-related factor 2; CT, computed tomography; GCS, Glasgow Coma Scale.

At post-injury 180 days, GOSE scores of patients ranged from 1 to 8 (median, 5; lower–upper quartiles, 2–6), and a total of 26, 9, 6, 8, 23, 16, 10, and 7 patients showed GOSE scores 1, 2, 3, 4, 5, 6, 7, and 8, respectively. In Figure 6, serum Nrf2 levels were markedly and inversely correlated with GOSE scores (*P* < 0.001), and patients with GOSE 1 had significantly highest serum Nrf2 levels and those with GOSE 8 displayed substantially lowest serum Nrf2 levels (*P* < 0.001). In aggregate, 49 patients had the development of a poor outcome (46.7%) at 180 days after trauma. In Table 4, as compared to patients with good outcome, poor outcome patients were prone to exhibit markedly increased proportion of diabetes mellitus, tended to show significantly decreased GCS scores, and were likely to exhibit substantially raised Rotterdam CT score, blood glucose levels, and serum Nrf2 levels. Furthermore, using the binary logistic regression model which contained the preceding variables, the factors independently associated with 180-day poor outcome were GCS scores, Rotterdam CT scores, and serum Nrf2 levels (all *P* < 0.05, Figure 7). Also, serum Nrf2 levels substantially discriminated patients at risk of 180-day poor outcome. Utilizing Youden's method, an optimal value of serum Nrf2 levels was chosen, which yielded medium–high sensitivity and specificity values for prediction of a poor outcome at 180 days after head trauma (Figure 7). Alternatively, its predictive capability for poor outcome was similar to those of GCS scores (AUC, 0.837; 95% CI, 0.767–0.907; *P* = 0.299) and Rotterdam CT scores (AUC, 0.822; 95% CI, 0.751–0.893; *P* = 0.544). Serum Nrf2 levels combined with GCS scores and Rotterdam CT scores (AUC, 0.889; 95% CI, 0.831–0.948) had significantly higher AUC than GCS scores (*P* = 0.035), Rotterdam CT scores (*P* = 0.006), or serum Nrf2 levels (*P* = 0.008) alone.

**Table 4:** Factors associated with 180-day poor outcome after severe traumatic brain injury.

	**Poor outcome**	**Good outcome**	***P-*value**
Gender (male/female)	28/21	32/24	1.000
Age (y)	45.0 ± 15.1	39.9 ± 13.0	0.070
Current cigarette smoking	19 (38.8%)	13 (23.2%)	0.084
Alcohol abuse	16 (32.7%)	17 (30.4%)	0.800
Hypertension	11 (22.5%)	6 (10.7%)	0.103
Diabetes mellitus	9 (18.4%)	3 (5.4%)	0.037
Hyperlipidemia	9 (18.4%)	8 (14.3%)	0.571
Hospital admission time (h)	4.7 (3.1–6.2)	5.1 (4.0–6.5)	0.182
Blood-sampling time (h)	6.2 (4.0–7.9)	6.8 (5.1–8.3)	0.203
Traumatic causes			0.351
Automobile/motorcycle	25	27	
Fall/jump	18	26	
Others	6	3	
GCS scores	4 (3–5)	5 (5–7)	< 0.001
Rotterdam CT scores	5 (4–6)	4 (3–4)	< 0.001
Systolic arterial pressure (mmHg)	122.9 ± 31.0	128.2 ± 28.2	0.356
Diastolic arterial pressure (mmHg)	74.8 ± 15.9	73.5 ± 18.6	0.713
Blood glucose levels (mmol/l)	9.8 (8.0–13.3)	8.2 (6.5–10.6)	0.011
Blood leucocyte count ( × 10^9^/l)	8.5 (6.2–10.9)	7.1 (5.6–9.3)	0.159
Serum Nrf2 levels (ng/ml)	12.5 (10.9–14.8)	9.4 (7.7–12.0)	< 0.001

Data were shown as mean ± standard deviation, median (25–75th percentiles), or count (percentage) where appropriate. Statistical methods for intergroup comparison included the t-test, Mann–Whitney U test, Pearson Chi-square test, or Fisher's exact test as appropriate. CT, computerized tomography; GCS, Glasgow Coma Scale; Nrf2, nuclear factor erythroid 2-related factor 2.

## Discussion

To the best of our knowledge, this study, for the first time, measured Nrf2 levels in the peripheral blood of patients with acute brain injury and further discerned the relationship between serum Nrf2 levels and illness severity plus functional outcome after sTBI. The main findings of our study are that (1) sTBI patients had significantly higher serum Nrf2 levels than healthy controls; (2) serum Nrf2 levels were in independent correlation with GCS scores and Rotterdam CT scores; (3) serum Nrf2 was an independent predictor of 180-day death, overall survival, and poor outcome; and (4) serum Nrf2 levels were in possession of significant prognostic predictive ability. Assumably, serum Nrf2 levels may be highly correlated with trauma severity and be tightly associated with a long-term functional outcome after sTBI, indicating that serum Nrf2 should represent a potential prognostic biochemical marker of sTBI.

Oxidative stress harbors an important role in some pathophysiologic processes that emerge following acute brain injury, including TBI (16). As a fact, oxidative stress appears when there is a disbalance between antioxidants and oxidants (17). Reactive oxygen species (ROS) belong to oxidants (18). ROS damage permeability of blood–brain barrier, promote vascular and cellular brain edema, and result in occurrence and progression of neuroinflammation after TBI (19). Nrf2 is identified as a new transcription factor which can regulate a set of ARE-dependent gene (20). After exposure to oxidative stress, Nrf2 translocates to the nucleus, activates ARE-dependent gene expression, and thereby leads to upregulation of expressions of various detoxification and antioxidant enzymes, such as superoxide dismutase, glutathione, and thioredoxin (21). In some experimental studies, Nrf2 knockout obviously increased production of ROS, aggravated brain injury, and worsened neurological function of animals with acute brain injury, including TBI (10–13), thus supporting the notion that Nrf2 may confer brain-protective function *via* reducing oxidative damage.

Nuclear factor erythroid 2-related factor 2 was greatly expressed in mouse brain after transient middle cerebral artery occlusion (11) and in brain of rats with intracerebral hemorrhage (22). Also, the expression of Nrf2 was obviously upregulated in brain tissues of patients with cerebral cortex contusion (14). Clearly, Nrf2 is located in glial cells and neuronal cells (11, 14, 22). Our study showed that serum Nrf2 levels were markedly higher in patients with sTBI than in healthy controls. Collectively, Nrf2 in the peripheral blood may be at least partially derived from injured brain tissues after sTBI. Given that Nrf2 may be protective against oxidative damage in animal experiments (10–13), Nrf2 expressions is hypothesized to be upregulated in response to brain oxidative injury after sTBI.

In the current study, in order to assess relationship between serum Nrf2 levels and trauma severity in addition to long-term prognosis, GCS and Rotterdam CT classification were selected as the two indicators of trauma severity, and 180-day GOSE scores and death were regarded as prognostic parameters. As regards statistical methods, multiple multivariate models were configured. Our data showed that serum Nrf2 levels were in independent correlation with trauma severity reflected by GCS scores and Rotterdam CT scores and were independently predictive of death, overall survival, and poor prognosis at 180 days after head trauma. Meanwhile, its prognostic predictive capability was confirmed under ROC curve. Intriguingly, serum Nrf2 levels combined with GCS scores and Rotterdam CT scores for death or poor outcome prediction displayed significantly higher AUC than GCS scores, Rotterdam CT scores, or serum Nrf2 levels alone. In summary, serum Nrf2 may serve as a promising prognostic biomarker.

There are several limitations in this study. First, the blood of sTBI patients was collected at a median value of 6.3 h after trauma. No treatments for glycemic control had been done, and serum Nrf2 levels were not significantly correlated with blood glucose levels in this study. However, Nrf2 signaling pathways regulation was associated with the hypoglycemic effect of specific agents and hypoglycemia-induced blood–brain barrier endothelial dysfunction *in vitro* (23). Thus, it would be very interesting to study whether glycemic control may affect serum Nrf2 levels after head trauma. Second, some treatments or complications, such as emergency operation, hypoxemia, and shock, may be associated with prognosis of sTBI (1) and therefore are considered as the confounding factors. In this study, the preceding factors are not investigated, and consequently, it will be significant for supplementing such variables in outcome analysis in future. Third, 180-day or 6-month neurological functional status is very commonly used to assess clinical outcome of patients with sTBI (24, 25). Length of ICU stay is sometimes selected as a prognostic parameter in the outcome study of head trauma (26, 27). Hence, it may be of clinical value that length of ICU stay is determined with respect to outcome analysis of human TBI. Last, there is an obvious difference in Nrf2 serum levels published values (28, 29) and values reported in this study. In the current study, we used the DNA-binding ELISA kit, which is in use in another clinical study (30) and is different from that utilized in previous studies (28, 29). So, differences in terms of serum Nrf2 levels between this study and others (28, 29) may be caused by different ELISA kits. However, it is necessary that values of serum Nrf2 levels can be validated using at least two sorts of ELISA kits in future.

## Conclusion

To the best of my knowledge, our study, for the first time, showed that increased serum Nrf2 levels of sTBI patients are independently correlated with GCS scores and Rotterdam CT scores and were independently predictive of 180-day death, overall survival, and poor outcome. Moreover, serum Nrf2 levels combined with GCS scores and Rotterdam CT scores for death or poor outcome prediction had significantly higher discriminatory efficiency than any one of them. Thus, it is hypothesized that serum Nrf2 may represent a potential prognostic biomarker of sTBI.

## Data availability statement

The raw data supporting the conclusions of this article will be made available by the authors, without undue reservation.

## Ethics statement

The studies involving human participants were reviewed and approved by the Quzhou Affiliated Hospital of Wenzhou Medical University. The patients/participants provided their written informed consent to participate in this study.

## Author contributions

All authors listed have made a substantial, direct, and intellectual contribution to the work and approved it for publication.

## Conflict of interest

The authors declare that the research was conducted in the absence of any commercial or financial relationships that could be construed as a potential conflict of interest.

## Publisher's note

All claims expressed in this article are solely those of the authors and do not necessarily represent those of their affiliated organizations, or those of the publisher, the editors and the reviewers. Any product that may be evaluated in this article, or claim that may be made by its manufacturer, is not guaranteed or endorsed by the publisher.

## Abbreviations

CT: computerized tomography
GCS: Glasgow Coma Scale
ROC: receiver operating characteristic
sTBI: severe traumatic brain injury
ROC: receiver operating characteristic
AUC: area under curve
OR: odds ratio
HR: hazard ratio
ROS: reactive oxygen species
Nrf2: nuclear factor erythroid 2-related factor 2
ARE: antioxidant response element
GOSE: extended Glasgow Outcome Scale.
